# Carbonyl[4-(2,6-dimethyl­phenyl­amino)pent-3-en-2-onato-κ^2^ *N*,*O*](triphenyl­phosphine-κ*P*)rhodium(I) acetone hemi­solvate

**DOI:** 10.1107/S160053680904817X

**Published:** 2009-11-18

**Authors:** Gertruida J. S. Venter, Gideon Steyl, Andreas Roodt

**Affiliations:** a Department of Chemistry, University of the Free State, P.O. Box 339, Bloemfontein, 9300, South Africa

## Abstract

In the title compound, [Rh(C_13_H_16_NO)(C_18_H_15_P)(CO)]·0.5C_3_H_6_O, the Rh atom exhibits a square-planar coordination geometry, being coordinated by the N and O atoms of the bidentate β-diketonato ligand, a P atom from the triphenyl­phosphine unit and a C atom from the carbonyl group. The asymmetric unit also contains a disordered half-mol­ecule, lying about an inversion center, of the acetone solvate. Inter­molecular C—H⋯O hydrogen bonds are observed between a C—H group of the triphenyl­phosphine unit and a carbonyl O atom and between the methyl group of the enamino­ketonato backbone and the solvent O atom. In addition, an intra­molecular inter­action is observed between a C—H group of the triphenyl­phosphine unit and the O atom of the enamino­ketonato ligand.

## Related literature

For synthetic background, see: Shaheen *et al.* (2006 ▶); Cornils & Hermann (1996); Bonati & Wilkinson (1964 ▶). For appplications of rhodium(I) dicarbonyl complexes, see: Cornils & Herrmann (1996 ▶); Trzeciak & Ziolkowski (1994 ▶); Van Rooy *et al.* (1995 ▶). For related structures, see: Damoense *et al.* (1994 ▶); Varshavsky *et al.* (2001 ▶); Venter *et al.* (2009 ▶).
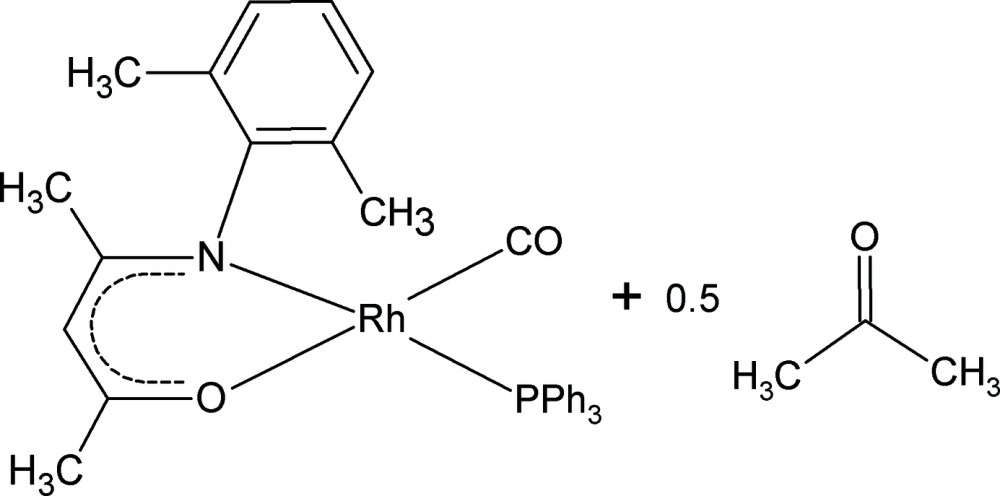


## Experimental

### 

#### Crystal data

[Rh(C_13_H_16_NO)(C_18_H_15_P)(CO)]·0.5C_3_H_6_O
*M* *_r_* = 624.5
Monoclinic, 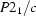

*a* = 16.8558 (8) Å
*b* = 11.4028 (5) Å
*c* = 16.4059 (8) Å
β = 108.733 (1)°
*V* = 2986.2 (2) Å^3^
*Z* = 4
Mo *K*α radiation
μ = 0.66 mm^−1^
*T* = 100 K
0.31 × 0.15 × 0.11 mm

#### Data collection

Bruker X8 APEXII 4K Kappa CCD diffractometer
Absorption correction: multi-scan (*SADABS*; Bruker, 2004 ▶) *T*_min_ = 0.822, *T*_max_ = 0.931
23158 measured reflections
7425 independent reflections
6081 reflections with > 2σ*I*)
*R*_int_ = 0.042

#### Refinement

*R*[*F* ^2^ > 2σ(*F* ^2^)] = 0.038
*wR*(*F* ^2^) = 0.112
*S* = 1.09
7425 reflections
354 parameters
1 restraint
H-atom parameters constrained
Δρ_max_ = 1.54 e Å^−3^
Δρ_min_ = −1.32 e Å^−3^

### 

Data collection: *APEX2* (Bruker, 2005 ▶); cell refinement: *SAINT-Plus* (Bruker, 2004 ▶); data reduction: *SAINT-Plus*; program(s) used to solve structure: *SIR97* (Altomare *et al.*, 1999 ▶); program(s) used to refine structure: *SHELXL97* (Sheldrick, 2008 ▶); molecular graphics: *DIAMOND* (Brandenburg & Putz, 2005 ▶); software used to prepare material for publication: *WinGX* (Farrugia, 1999 ▶).

## Supplementary Material

Crystal structure: contains datablocks global, I. DOI: 10.1107/S160053680904817X/pv2227sup1.cif

Structure factors: contains datablocks I. DOI: 10.1107/S160053680904817X/pv2227Isup2.hkl

Additional supplementary materials:  crystallographic information; 3D view; checkCIF report

## Figures and Tables

**Table 1:** Hydrogen-bond geometry (Å, °)

*D*—H⋯*A*	*D*—H	H⋯*A*	*D*⋯*A*	*D*—H⋯*A*
C332—H332⋯O12	0.95	2.38	3.201 (3)	144
C334—H334⋯O14^i^	0.95	2.51	3.201 (4)	130
C1—H1*B*⋯O01^ii^	0.98	2.54	3.372 (9)	142

Symmetry codes: (i) 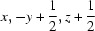; (ii) 
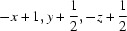
.

**Table 2:** Comparative geometrical parameters (Å, °) for similar [Rh(*N*,*O*-bid)(CO)(PPh_3_)] complexes

Parameters	(I)^*a*^	(II)^*b*^	(III)^*c*^	(IV)^*d*^
Rh1—N11	2.077 (2)	2.069 (2)	2.045 (4)	2.045 (3)
Rh1—O12	2.027 (2)	2.028 (2)	2.044 (3)	2.045 (2)
Rh1—P13	2.2704 (7)	2.2635 (6)	2.275 (1)	2.281 (2)
Rh1—C14	1.812 (3)	1.807 (2)	1.784 (5)	1.804 (3)
C14—O14	1.147 (3)	1.152 (3)	1.142 (7)	1.148 (4)
N11⋯O12	2.885 (3)	2.885 (3)	2.826 (6)	2.841 (3)
N11—Rh1—O12	89.31 (9)	89.54 (8)	87.4 (1)	87.95 (8)
O12—Rh1—P13	85.95 (6)	84.97 (5)	89.7 (1)	89.91 (5)
P13—Rh1—C14	91.57 (9)	91.87 (7)	90.3 (2)	89.48 (9)
N11—Rh1—C14	93.1 (1)	93.6 (1)	92.6 (2)	92.6 (1)
N11—C2—C4—O12	−2.6 (2)	4.1 (2)	1.2 (4)	1.5 (2)
θ_E_^*e*^	155.77 (2)	156.39 (3)	156.0 (2)	156.23 (4)

Notes: (*a*) This work; (*b*) *N*,*O*-bid = 4-(2,3-dimethyl phenylamino)pent-3-en-2-onato (Venter *et al.*, 2009 ▶); (*c*) *N*,*O*-bid = 4-amino-pent-3-en-2-onato (Damoense *et al.*, 1994 ▶); (*d*) *N*,*O*-bid = 4-amino-1,1,1-trifluoro-pent-3-en-2-onato (Varshavsky *et al.*, 2001 ▶); (*e*) cone angle (Tolman, 1977 ▶).

**Fig. 1.**
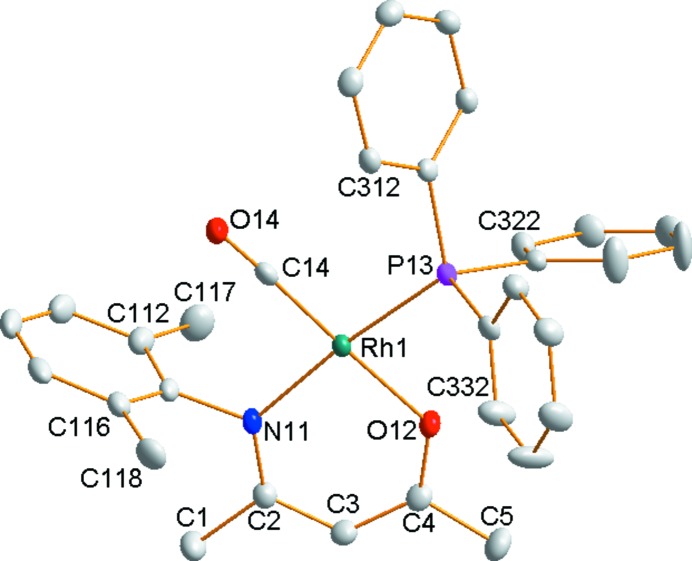
Molecular structure of the title compound. Displacement ellipsoids are drawn at the 50% probability displacement level. H atoms and disordered solvate molecule have been omitted for clarity.

**Fig. 2.**
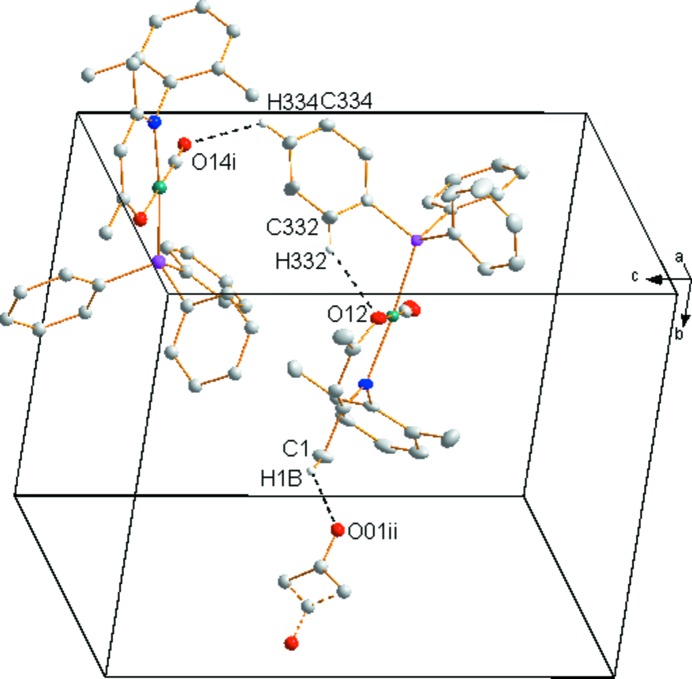
A view of the unit cell of (I) illustrating the C—H···O interactions; hydrogen atoms have been omitted for clarity.

## supplementary crystallographic information

### Comment

Rhodium(I) dicarbonyl complexes of the type [Rh(*L*,*L*')(CO)_2_] containing chelating mono-anionic bidentate (*L*,*L*') ligands coordinated to rhodium *via* (O,*O*) donor atoms have been studied as catalyst precursors (Cornils & Herrmann, 1996; Trzeciak *et al.*, 1994; Van Rooy *et al.*, 1995). The investigation of these complexes is followed by complexes containing bidentate β-enaminoketonato ligands such as 4-(phenylamino)pent-3-en-2-onato (Phony) (Shaheen *et al.*, 2006) coordinated to rhodium *via* (*N*,*O*) donor atoms. It was suggested that only one CO-group in a [Rh(*N*,*O*-bid)(CO)_2_]-type complex will be substituted by triphenyl phosphine, with the product being one of two possible isomers (Bonati & Wilkinson, 1964). Since the N-donor atom has a larger *trans*-influence than the O-atom, the CO-group *trans* to the N-atom will be substituted. This is evident in the title compound, (I), where [Rh(2,6-diMe-Phony)(CO)(PPh_3_)] is formed by the substitution of the carbonyl ligand in the dicarbonyl rhodium(I) complex [Rh(2,6-diMe-Phony)(CO)_2_] by PPh_3_.

In the title complex (Fig. 1), the bond distances involving rhodium differ significantly from the distances in related complexes, with the exception of [Rh(2,3-diMe-Phony)(CO)(PPh_3_)] (Venter *et al.*, 2009), wherein all angles and distances are comparable to (I) (Table 2). The distance, Rh—N, in (I) is longer than in similar complexes while the Rh—O bond distance is shorter. This is due to the steric influence of the phenyl group connected to nitrogen in the title compound, as opposed to the hydrogen in the related complexes. The Rh—C and the carbonyl C—O distances are comparable with those distances in the related complexes (Table 2). The N—Rh—O bite angle is slightly larger than that observed in similar complexes found in the literature. The effective cone angle, θ_E_, (Tolman, 1977) of 155.77 (2)° is similar to the angles in the related compounds. The acetone solvate was disordered and was located about an inversion center. Intermolecular and intramolecular hydrogen bonds of the type C—H···O are observed in the structure.

### Experimental

To a 5 ml acetone solution of [Rh(2,6-diMe-Phony)(CO)_2_] (0.0302 g, 83.61 µmol) was added PPh_3_ (0.0219 g, 83.50 µmol) resulting in an immediate evolution of gas with the formation of the title compound. Crystallization from acetone produced yellow crystals of (I) in quantitative (0.0516 g, 100%) yield. IR (KBr): ν_CO_ 1971.1 s cm^-1^.

### Refinement

The methyl and aromatic H atoms were placed in geometrically idealized positions and constrained to ride on their parent atoms, with C—H = 0.95 and 0.98 Å and *U*_iso_(H) = 1.5*U*_eq_(C) and 1.2*U*_eq_(C), respectively. The methyl groups were generated to fit the difference electron density and the groups were then refined as rigid rotors. The acetone solvate was disordered about inversion center. The highest residual electron- density peak in the final difference map was located 0.65Å from H316 and was essentially meaningless.

### Crystal data

**Table:**

[Rh(C_13_H_16_NO)(C_18_H_15_P)(CO)]·0.5C_3_H_6_O	*F*(000) = 1288
*M**_r_* = 624.5	*D*_x_ = 1.389 Mg m^−^^3^
Monoclinic, *P*2_1_/*c*	Mo *K*α radiation, λ = 0.71073 Å
Hall symbol: -P 2ybc	Cell parameters from 6670 reflections
*a* = 16.8558 (8) Å	θ = 2.5–28.2°
*b* = 11.4028 (5) Å	µ = 0.66 mm^−^^1^
*c* = 16.4059 (8) Å	*T* = 100 K
β = 108.733 (1)°	Cuboid, yellow
*V* = 2986.2 (2) Å^3^	0.31 × 0.15 × 0.11 mm
*Z* = 4	

### Data collection

**Table:**

Bruker X8 APEXII 4K Kappa CCD diffractometer	7425 independent reflections
Radiation source: fine-focus sealed tube	6081 reflections with > 2σ*I*)
graphite	*R*_int_ = 0.042
ω and φ scans	θ_max_ = 28.3°, θ_min_ = 1.3°
Absorption correction: multi-scan (*SADABS*; Bruker, 2004)	*h* = −21→22
*T*_min_ = 0.822, *T*_max_ = 0.931	*k* = −15→11
23158 measured reflections	*l* = −21→21

### Refinement

**Table:**

Refinement on *F*^2^	Primary atom site location: structure-invariant direct methods
Least-squares matrix: full	Secondary atom site location: difference Fourier map
*R*[*F*^2^ > 2σ(*F*^2^)] = 0.038	Hydrogen site location: inferred from neighbouring sites
*wR*(*F*^2^) = 0.112	H-atom parameters constrained
*S* = 1.09	*w* = 1/[σ^2^(*F*_o_^2^) + (0.0577*P*)^2^ + 1.4787*P*] where *P* = (*F*_o_^2^ + 2*F*_c_^2^)/3
7425 reflections	(Δ/σ)_max_ = 0.002
354 parameters	Δρ_max_ = 1.54 e Å^−^^3^
1 restraint	Δρ_min_ = −1.32 e Å^−^^3^

### Special details

**Table:**

Experimental. The intensity data was collected on a Bruker X8 ApexII 4 K Kappa CCD diffractometer using an exposure time of 60 s/frame. A total of 688 frames were collected with a frame width of 0.5° covering up to θ = 28.24° with 99.8% completeness accomplished.
Geometry. All e.s.d.'s (except the e.s.d. in the dihedral angle between two l.s. planes) are estimated using the full covariance matrix. The cell e.s.d.'s are taken into account individually in the estimation of e.s.d.'s in distances, angles and torsion angles; correlations between e.s.d.'s in cell parameters are only used when they are defined by crystal symmetry. An approximate (isotropic) treatment of cell e.s.d.'s is used for estimating e.s.d.'s involving l.s. planes.

### Fractional atomic coordinates and isotropic or equivalent isotropic displacement parameters (Å^2^)

**Table:**

	*x*	*y*	*z*	*U*_iso_*/*U*_eq_	Occ. (<1)
C1	0.7144 (2)	0.7625 (3)	0.4744 (2)	0.0257 (7)	
H1A	0.7697	0.7807	0.5152	0.039*	
H1B	0.6712	0.7804	0.5007	0.039*	
H1C	0.7048	0.8097	0.4222	0.039*	
C2	0.71032 (17)	0.6337 (2)	0.45128 (18)	0.0168 (5)	
C3	0.65757 (17)	0.5637 (2)	0.48382 (18)	0.0183 (6)	
H3	0.6324	0.6017	0.5209	0.022*	
C4	0.63880 (17)	0.4461 (2)	0.46740 (19)	0.0180 (6)	
C5	0.57863 (19)	0.3856 (3)	0.5045 (2)	0.0265 (7)	
H5A	0.5288	0.3609	0.4577	0.04*	
H5B	0.5621	0.4399	0.5425	0.04*	
H5C	0.6058	0.3167	0.5375	0.04*	
C14	0.84522 (17)	0.4446 (2)	0.32802 (17)	0.0166 (5)	
C111	0.80357 (17)	0.6734 (2)	0.37352 (18)	0.0164 (5)	
C112	0.76869 (19)	0.7250 (2)	0.2919 (2)	0.0223 (6)	
C113	0.8183 (2)	0.8000 (3)	0.2624 (2)	0.0271 (7)	
H113	0.7952	0.8365	0.2078	0.033*	
C114	0.9008 (2)	0.8226 (2)	0.3109 (2)	0.0292 (7)	
H114	0.9338	0.8746	0.2897	0.035*	
C115	0.93503 (19)	0.7690 (2)	0.3902 (2)	0.0242 (6)	
H115	0.9919	0.7836	0.4228	0.029*	
C116	0.88743 (18)	0.6938 (2)	0.42310 (19)	0.0194 (6)	
C117	0.6798 (2)	0.6980 (3)	0.2380 (2)	0.0318 (7)	
H11A	0.6667	0.7385	0.1826	0.048*	
H11B	0.6414	0.7247	0.2682	0.048*	
H11C	0.6734	0.6132	0.2283	0.048*	
C118	0.92484 (19)	0.6332 (3)	0.5082 (2)	0.0257 (7)	
H11D	0.9859	0.6413	0.5269	0.039*	
H11E	0.9099	0.5499	0.5021	0.039*	
H11F	0.9031	0.6689	0.551	0.039*	
C311	0.82259 (17)	0.1691 (2)	0.27715 (17)	0.0149 (5)	
C312	0.90839 (17)	0.1910 (2)	0.30147 (18)	0.0168 (5)	
H312	0.9352	0.2308	0.354	0.02*	
C313	0.95533 (17)	0.1553 (2)	0.24967 (19)	0.0193 (6)	
H313	1.0138	0.1707	0.267	0.023*	
C314	0.91660 (18)	0.0976 (2)	0.17315 (19)	0.0212 (6)	
H314	0.9483	0.0736	0.1375	0.025*	
C315	0.83113 (18)	0.0749 (2)	0.14845 (19)	0.0190 (6)	
H315	0.8045	0.035	0.0959	0.023*	
C316	0.78449 (17)	0.1103 (2)	0.20019 (18)	0.0168 (5)	
H316	0.7261	0.0943	0.1829	0.02*	
C321	0.66208 (17)	0.1553 (2)	0.30122 (17)	0.0153 (5)	
C322	0.59944 (17)	0.2176 (2)	0.24164 (19)	0.0202 (6)	
H322	0.6093	0.296	0.2279	0.024*	
C323	0.52197 (18)	0.1657 (3)	0.2017 (2)	0.0243 (6)	
H323	0.4799	0.208	0.1594	0.029*	
C324	0.50573 (19)	0.0534 (3)	0.2231 (2)	0.0260 (7)	
H324	0.4523	0.019	0.1966	0.031*	
C325	0.5676 (2)	−0.0085 (3)	0.2829 (2)	0.0386 (9)	
H325	0.557	−0.086	0.2979	0.046*	
C326	0.6459 (2)	0.0424 (3)	0.3217 (2)	0.0335 (8)	
H326	0.6884	−0.001	0.3627	0.04*	
C331	0.81170 (17)	0.1399 (2)	0.44688 (17)	0.0156 (5)	
C332	0.7927 (2)	0.1752 (3)	0.51929 (19)	0.0266 (7)	
H332	0.7579	0.2416	0.5163	0.032*	
C333	0.8240 (2)	0.1144 (3)	0.5959 (2)	0.0334 (8)	
H333	0.8091	0.1378	0.6447	0.04*	
C334	0.8770 (2)	0.0196 (3)	0.60188 (19)	0.0263 (7)	
H334	0.8999	−0.0205	0.655	0.032*	
C335	0.89635 (18)	−0.0163 (2)	0.53013 (19)	0.0222 (6)	
H335	0.932	−0.082	0.5339	0.027*	
C336	0.86407 (18)	0.0429 (2)	0.45242 (19)	0.0196 (6)	
H336	0.8775	0.0176	0.4032	0.024*	
N11	0.75420 (14)	0.59212 (18)	0.40401 (15)	0.0148 (5)	
O12	0.66821 (11)	0.37910 (16)	0.42069 (13)	0.0174 (4)	
O14	0.90058 (13)	0.46375 (18)	0.30306 (13)	0.0225 (4)	
P13	0.76530 (4)	0.22182 (6)	0.34722 (4)	0.01315 (15)	
Rh1	0.761010 (12)	0.417429 (17)	0.371227 (13)	0.01263 (8)	
O01	0.4759 (6)	0.3631 (7)	0.0331 (6)	0.0963 (16)	0.5
C01	0.4182 (4)	0.5252 (5)	−0.0389 (4)	0.0963 (16)	
H01A	0.366	0.4813	−0.0482	0.144*	
H01B	0.4236	0.5472	−0.0946	0.144*	
H01C	0.4173	0.5961	−0.0055	0.144*	
C02	0.4899 (9)	0.4513 (10)	0.0086 (9)	0.0963 (16)	0.5

### Atomic displacement parameters (Å^2^)

**Table:**

	*U*^11^	*U*^22^	*U*^33^	*U*^12^	*U*^13^	*U*^23^
C1	0.0290 (16)	0.0158 (14)	0.0378 (18)	0.0010 (12)	0.0183 (14)	−0.0024 (13)
C2	0.0145 (13)	0.0149 (13)	0.0204 (14)	0.0019 (10)	0.0049 (11)	0.0014 (10)
C3	0.0165 (13)	0.0185 (13)	0.0215 (14)	0.0041 (11)	0.0082 (11)	0.0009 (11)
C4	0.0127 (13)	0.0192 (13)	0.0220 (14)	0.0039 (10)	0.0056 (11)	0.0037 (11)
C5	0.0233 (15)	0.0239 (15)	0.0378 (18)	−0.0002 (12)	0.0176 (14)	0.0045 (13)
C14	0.0190 (14)	0.0134 (12)	0.0157 (13)	−0.0017 (10)	0.0032 (11)	−0.0036 (10)
C111	0.0206 (14)	0.0097 (12)	0.0224 (14)	−0.0009 (10)	0.0118 (12)	−0.0016 (10)
C112	0.0273 (15)	0.0152 (13)	0.0279 (16)	0.0027 (11)	0.0137 (13)	0.0022 (11)
C113	0.0408 (19)	0.0149 (13)	0.0312 (17)	0.0020 (13)	0.0193 (15)	0.0041 (12)
C114	0.0410 (19)	0.0138 (14)	0.043 (2)	−0.0063 (13)	0.0286 (16)	−0.0043 (13)
C115	0.0243 (15)	0.0173 (14)	0.0375 (18)	−0.0055 (11)	0.0189 (14)	−0.0117 (12)
C116	0.0220 (14)	0.0136 (12)	0.0242 (15)	−0.0014 (11)	0.0099 (12)	−0.0038 (11)
C117	0.0293 (17)	0.0335 (17)	0.0289 (17)	0.0046 (14)	0.0043 (14)	0.0104 (14)
C118	0.0217 (15)	0.0267 (16)	0.0243 (16)	−0.0035 (12)	0.0013 (13)	−0.0047 (12)
C311	0.0186 (13)	0.0098 (12)	0.0171 (13)	0.0012 (10)	0.0066 (11)	0.0014 (10)
C312	0.0165 (13)	0.0147 (12)	0.0180 (13)	−0.0017 (10)	0.0037 (11)	−0.0003 (10)
C313	0.0138 (13)	0.0189 (13)	0.0249 (15)	0.0030 (11)	0.0059 (11)	0.0037 (11)
C314	0.0225 (15)	0.0196 (14)	0.0242 (15)	0.0043 (11)	0.0112 (13)	0.0025 (11)
C315	0.0211 (14)	0.0176 (13)	0.0180 (14)	−0.0002 (11)	0.0060 (12)	−0.0023 (10)
C316	0.0165 (13)	0.0143 (12)	0.0187 (14)	−0.0012 (10)	0.0041 (11)	−0.0001 (10)
C321	0.0159 (13)	0.0149 (12)	0.0163 (13)	−0.0011 (10)	0.0067 (11)	−0.0025 (10)
C322	0.0173 (14)	0.0168 (13)	0.0251 (15)	−0.0012 (11)	0.0049 (12)	−0.0008 (11)
C323	0.0155 (14)	0.0265 (16)	0.0261 (16)	0.0030 (12)	0.0001 (12)	0.0001 (12)
C324	0.0166 (14)	0.0312 (16)	0.0283 (16)	−0.0067 (12)	0.0045 (13)	−0.0052 (13)
C325	0.0317 (18)	0.0261 (17)	0.048 (2)	−0.0167 (14)	−0.0006 (16)	0.0098 (15)
C326	0.0249 (16)	0.0237 (16)	0.041 (2)	−0.0047 (13)	−0.0047 (15)	0.0129 (14)
C331	0.0162 (13)	0.0134 (12)	0.0147 (13)	−0.0016 (10)	0.0014 (11)	−0.0017 (10)
C332	0.0368 (18)	0.0224 (15)	0.0195 (15)	0.0132 (13)	0.0077 (13)	0.0011 (12)
C333	0.054 (2)	0.0313 (17)	0.0153 (15)	0.0234 (16)	0.0113 (15)	0.0034 (13)
C334	0.0340 (17)	0.0214 (15)	0.0188 (15)	0.0074 (13)	0.0022 (13)	0.0031 (12)
C335	0.0246 (15)	0.0168 (14)	0.0233 (15)	0.0049 (11)	0.0052 (12)	0.0005 (11)
C336	0.0220 (14)	0.0160 (13)	0.0213 (14)	0.0013 (11)	0.0075 (12)	−0.0017 (11)
N11	0.0142 (11)	0.0107 (10)	0.0191 (12)	−0.0009 (8)	0.0049 (9)	0.0012 (8)
O12	0.0152 (9)	0.0148 (9)	0.0242 (10)	−0.0008 (8)	0.0091 (8)	0.0014 (8)
O14	0.0261 (11)	0.0180 (10)	0.0280 (11)	−0.0059 (8)	0.0152 (9)	−0.0034 (8)
P13	0.0123 (3)	0.0117 (3)	0.0142 (3)	−0.0008 (2)	0.0027 (3)	−0.0002 (2)
Rh1	0.01250 (12)	0.01060 (11)	0.01490 (12)	−0.00047 (7)	0.00457 (8)	0.00021 (7)
O01	0.139 (5)	0.057 (3)	0.104 (4)	−0.010 (3)	0.054 (4)	0.007 (2)
C01	0.139 (5)	0.057 (3)	0.104 (4)	−0.010 (3)	0.054 (4)	0.007 (2)
C02	0.139 (5)	0.057 (3)	0.104 (4)	−0.010 (3)	0.054 (4)	0.007 (2)

### Geometric parameters (Å, °)

**Table:**

C1—C2	1.513 (4)	C313—H313	0.95
C1—H1A	0.98	C314—C315	1.390 (4)
C1—H1B	0.98	C314—H314	0.95
C1—H1C	0.98	C315—C316	1.389 (4)
C2—N11	1.320 (4)	C315—H315	0.95
C2—C3	1.420 (4)	C316—H316	0.95
C3—C4	1.384 (4)	C321—C326	1.380 (4)
C3—H3	0.95	C321—C322	1.383 (4)
C4—O12	1.289 (3)	C321—P13	1.824 (3)
C4—C5	1.507 (4)	C322—C323	1.391 (4)
C5—H5A	0.98	C322—H322	0.95
C5—H5B	0.98	C323—C324	1.378 (4)
C5—H5C	0.98	C323—H323	0.95
C14—O14	1.154 (3)	C324—C325	1.375 (4)
C14—Rh1	1.805 (3)	C324—H324	0.95
C111—C116	1.405 (4)	C325—C326	1.394 (4)
C111—C112	1.406 (4)	C325—H325	0.95
C111—N11	1.439 (3)	C326—H326	0.95
C112—C113	1.387 (4)	C331—C332	1.385 (4)
C112—C117	1.507 (4)	C331—C336	1.400 (4)
C113—C114	1.388 (5)	C331—P13	1.827 (3)
C113—H113	0.95	C332—C333	1.383 (4)
C114—C115	1.385 (5)	C332—H332	0.95
C114—H114	0.95	C333—C334	1.386 (4)
C115—C116	1.396 (4)	C333—H333	0.95
C115—H115	0.95	C334—C335	1.380 (4)
C116—C118	1.503 (4)	C334—H334	0.95
C117—H11A	0.98	C335—C336	1.390 (4)
C117—H11B	0.98	C335—H335	0.95
C117—H11C	0.98	C336—H336	0.95
C118—H11D	0.98	N11—Rh1	2.076 (2)
C118—H11E	0.98	O12—Rh1	2.0277 (19)
C118—H11F	0.98	P13—Rh1	2.2701 (7)
C311—C316	1.391 (4)	O01—C02	1.135 (12)
C311—C312	1.394 (4)	C01—C02	1.474 (13)
C311—P13	1.825 (3)	C01—H01A	0.98
C312—C313	1.395 (4)	C01—H01B	0.98
C312—H312	0.95	C01—H01C	0.98
C313—C314	1.382 (4)	C02—C01^i^	1.492 (16)
			
C2—C1—H1A	109.5	C314—C315—H315	119.8
C2—C1—H1B	109.5	C315—C316—C311	120.5 (3)
H1A—C1—H1B	109.5	C315—C316—H316	119.7
C2—C1—H1C	109.5	C311—C316—H316	119.7
H1A—C1—H1C	109.5	C326—C321—C322	119.0 (3)
H1B—C1—H1C	109.5	C326—C321—P13	121.6 (2)
N11—C2—C3	123.8 (2)	C322—C321—P13	119.3 (2)
N11—C2—C1	120.5 (2)	C321—C322—C323	120.2 (3)
C3—C2—C1	115.8 (2)	C321—C322—H322	119.9
C4—C3—C2	127.1 (3)	C323—C322—H322	119.9
C4—C3—H3	116.5	C324—C323—C322	120.6 (3)
C2—C3—H3	116.5	C324—C323—H323	119.7
O12—C4—C3	125.6 (3)	C322—C323—H323	119.7
O12—C4—C5	113.9 (2)	C325—C324—C323	119.4 (3)
C3—C4—C5	120.4 (3)	C325—C324—H324	120.3
C4—C5—H5A	109.5	C323—C324—H324	120.3
C4—C5—H5B	109.5	C324—C325—C326	120.1 (3)
H5A—C5—H5B	109.5	C324—C325—H325	119.9
C4—C5—H5C	109.5	C326—C325—H325	119.9
H5A—C5—H5C	109.5	C321—C326—C325	120.6 (3)
H5B—C5—H5C	109.5	C321—C326—H326	119.7
O14—C14—Rh1	177.7 (2)	C325—C326—H326	119.7
C116—C111—C112	121.1 (3)	C332—C331—C336	119.2 (3)
C116—C111—N11	119.5 (2)	C332—C331—P13	117.9 (2)
C112—C111—N11	119.3 (2)	C336—C331—P13	122.8 (2)
C113—C112—C111	118.4 (3)	C333—C332—C331	120.5 (3)
C113—C112—C117	121.2 (3)	C333—C332—H332	119.7
C111—C112—C117	120.4 (3)	C331—C332—H332	119.7
C112—C113—C114	121.3 (3)	C332—C333—C334	120.3 (3)
C112—C113—H113	119.3	C332—C333—H333	119.9
C114—C113—H113	119.3	C334—C333—H333	119.9
C115—C114—C113	119.6 (3)	C335—C334—C333	119.6 (3)
C115—C114—H114	120.2	C335—C334—H334	120.2
C113—C114—H114	120.2	C333—C334—H334	120.2
C114—C115—C116	121.2 (3)	C334—C335—C336	120.5 (3)
C114—C115—H115	119.4	C334—C335—H335	119.7
C116—C115—H115	119.4	C336—C335—H335	119.7
C115—C116—C111	118.3 (3)	C335—C336—C331	119.8 (3)
C115—C116—C118	121.3 (3)	C335—C336—H336	120.1
C111—C116—C118	120.4 (3)	C331—C336—H336	120.1
C112—C117—H11A	109.5	C2—N11—C111	118.0 (2)
C112—C117—H11B	109.5	C2—N11—Rh1	125.79 (18)
H11A—C117—H11B	109.5	C111—N11—Rh1	116.24 (17)
C112—C117—H11C	109.5	C4—O12—Rh1	127.13 (18)
H11A—C117—H11C	109.5	C311—P13—C321	103.27 (12)
H11B—C117—H11C	109.5	C311—P13—C331	103.55 (12)
C116—C118—H11D	109.5	C321—P13—C331	103.59 (12)
C116—C118—H11E	109.5	C311—P13—Rh1	119.08 (9)
H11D—C118—H11E	109.5	C321—P13—Rh1	113.64 (9)
C116—C118—H11F	109.5	C331—P13—Rh1	112.06 (9)
H11D—C118—H11F	109.5	C14—Rh1—O12	177.36 (10)
H11E—C118—H11F	109.5	C14—Rh1—N11	93.02 (11)
C316—C311—C312	118.7 (3)	O12—Rh1—N11	89.38 (8)
C316—C311—P13	123.2 (2)	C14—Rh1—P13	91.59 (9)
C312—C311—P13	118.1 (2)	O12—Rh1—P13	85.95 (6)
C311—C312—C313	120.8 (3)	N11—Rh1—P13	174.36 (7)
C311—C312—H312	119.6	C02—C01—H01A	109.5
C313—C312—H312	119.6	C02—C01—H01B	109.5
C314—C313—C312	119.8 (3)	H01A—C01—H01B	109.5
C314—C313—H313	120.1	C02—C01—H01C	109.5
C312—C313—H313	120.1	H01A—C01—H01C	109.5
C313—C314—C315	119.8 (3)	H01B—C01—H01C	109.5
C313—C314—H314	120.1	O01—C02—C01	117.7 (13)
C315—C314—H314	120.1	O01—C02—C01^i^	110.6 (12)
C316—C315—C314	120.3 (3)	C01—C02—C01^i^	131.4 (9)
C316—C315—H315	119.8		
			
N11—C2—C3—C4	−4.8 (5)	P13—C331—C336—C335	−179.2 (2)
C1—C2—C3—C4	175.4 (3)	C3—C2—N11—C111	178.5 (3)
C2—C3—C4—O12	1.8 (5)	C1—C2—N11—C111	−1.7 (4)
C2—C3—C4—C5	−177.6 (3)	C3—C2—N11—Rh1	−3.0 (4)
C116—C111—C112—C113	−2.1 (4)	C1—C2—N11—Rh1	176.8 (2)
N11—C111—C112—C113	−177.7 (3)	C116—C111—N11—C2	91.5 (3)
C116—C111—C112—C117	177.3 (3)	C112—C111—N11—C2	−92.9 (3)
N11—C111—C112—C117	1.7 (4)	C116—C111—N11—Rh1	−87.2 (3)
C111—C112—C113—C114	1.2 (4)	C112—C111—N11—Rh1	88.5 (3)
C117—C112—C113—C114	−178.3 (3)	C3—C4—O12—Rh1	9.0 (4)
C112—C113—C114—C115	0.4 (5)	C5—C4—O12—Rh1	−171.64 (18)
C113—C114—C115—C116	−1.1 (4)	C316—C311—P13—C321	9.9 (3)
C114—C115—C116—C111	0.1 (4)	C312—C311—P13—C321	−171.4 (2)
C114—C115—C116—C118	178.5 (3)	C316—C311—P13—C331	117.7 (2)
C112—C111—C116—C115	1.5 (4)	C312—C311—P13—C331	−63.6 (2)
N11—C111—C116—C115	177.1 (2)	C316—C311—P13—Rh1	−117.1 (2)
C112—C111—C116—C118	−176.9 (3)	C312—C311—P13—Rh1	61.6 (2)
N11—C111—C116—C118	−1.3 (4)	C326—C321—P13—C311	83.9 (3)
C316—C311—C312—C313	0.3 (4)	C322—C321—P13—C311	−93.4 (2)
P13—C311—C312—C313	−178.5 (2)	C326—C321—P13—C331	−23.8 (3)
C311—C312—C313—C314	0.1 (4)	C322—C321—P13—C331	158.9 (2)
C312—C313—C314—C315	−0.3 (4)	C326—C321—P13—Rh1	−145.7 (2)
C313—C314—C315—C316	0.2 (4)	C322—C321—P13—Rh1	37.0 (2)
C314—C315—C316—C311	0.1 (4)	C332—C331—P13—C311	168.3 (2)
C312—C311—C316—C315	−0.4 (4)	C336—C331—P13—C311	−12.7 (3)
P13—C311—C316—C315	178.3 (2)	C332—C331—P13—C321	−84.2 (2)
C326—C321—C322—C323	−1.5 (4)	C336—C331—P13—C321	94.8 (3)
P13—C321—C322—C323	175.9 (2)	C332—C331—P13—Rh1	38.7 (3)
C321—C322—C323—C324	2.2 (5)	C336—C331—P13—Rh1	−142.3 (2)
C322—C323—C324—C325	−1.5 (5)	C4—O12—Rh1—N11	−11.8 (2)
C323—C324—C325—C326	0.2 (6)	C4—O12—Rh1—P13	165.0 (2)
C322—C321—C326—C325	0.2 (5)	C2—N11—Rh1—C14	−170.1 (2)
P13—C321—C326—C325	−177.2 (3)	C111—N11—Rh1—C14	8.5 (2)
C324—C325—C326—C321	0.5 (6)	C2—N11—Rh1—O12	8.8 (2)
C336—C331—C332—C333	−0.9 (5)	C111—N11—Rh1—O12	−172.62 (19)
P13—C331—C332—C333	178.1 (3)	C311—P13—Rh1—C14	−14.40 (13)
C331—C332—C333—C334	2.0 (5)	C321—P13—Rh1—C14	−136.42 (13)
C332—C333—C334—C335	−2.0 (5)	C331—P13—Rh1—C14	106.57 (13)
C333—C334—C335—C336	0.9 (5)	C311—P13—Rh1—O12	166.55 (12)
C334—C335—C336—C331	0.2 (4)	C321—P13—Rh1—O12	44.53 (11)
C332—C331—C336—C335	−0.2 (4)	C331—P13—Rh1—O12	−72.48 (11)

Symmetry codes: (i) −*x*+1, −*y*+1, −*z*.

### Hydrogen-bond geometry (Å, °)

**Table:**

*D*—H···*A*	*D*—H	H···*A*	*D*···*A*	*D*—H···*A*
C332—H332···O12	0.95	2.38	3.201 (3)	144
C334—H334···O14^ii^	0.95	2.51	3.201 (4)	130
C1—H1B···O01^iii^	0.98	2.54	3.372 (9)	142

Symmetry codes: (ii) *x*, −*y*+1/2, *z*+1/2; (iii) −*x*+1, *y*+1/2, −*z*+1/2.

### Table 2 Comparative geometrical parameters (Å, °) for similar [Rh(*N*,*O*-bid)(CO)(PPh_3_)] complexes

**Table:**

Parameters	(I)*^a^*	(II)*^b^*	(III)*^c^*	(IV)*^d^*
Rh1—N11	2.077 (2)	2.069 (2)	2.045 (4)	2.045 (3)
Rh1—O12	2.027 (2)	2.028 (2)	2.044 (3)	2.045 (2)
Rh1—P13	2.2704 (7)	2.2635 (6)	2.275 (1)	2.281 (2)
Rh1—C14	1.812 (3)	1.807 (2)	1.784 (5)	1.804 (3)
C14—O14	1.147 (3)	1.152 (3)	1.142 (7)	1.148 (4)
N11···O12	2.885 (3)	2.885 (3)	2.826 (6)	2.841 (3)
N11—Rh1—O12	89.31 (9)	89.54 (8)	87.4 (1)	87.95 (8)
O12—Rh1—P13	85.95 (6)	84.97 (5)	89.7 (1)	89.91 (5)
P13—Rh1—C14	91.57 (9)	91.87 (7)	90.3 (2)	89.48 (9)
N11—Rh1—C14	93.1 (1)	93.6 (1)	92.6 (2)	92.6 (1)
N11—C2—C4—O12	-2.6 (2)	4.1 (2)	1.2 (4)	1.5 (2)
θ_E_*^e^*	155.77 (2)	156.39 (3)	156.0 (2)	156.23 (4)

Notes: (*a*) This work; (*b*) *N*,*O*-bid = 4-(2,3-dimethyl phenylamino)pent-3-en-2-onato (Venter *et al.*, 2009); (*c*) *N*,*O*-bid = 4-amino-pent-3-en-2-onato (Damoense *et al.*, 1994); (*d*) *N*,*O*-bid = 4-amino-1,1,1-trifluoro-pent-3-en-2-onato (Varshavsky *et al.*, 2001); (*e*) Tolman (1977).
